# Pulmonary Embolism in a Patient with Dual Inferior Vena Cava

**DOI:** 10.1055/a-2826-4664

**Published:** 2026-05-16

**Authors:** Huaqiang Fang, Kanghui Dai, Xianhua Zhu, Zishan Deng, Jiehua Qiu

**Affiliations:** 1 196534 Department of Vascular Surgery, Second Affiliated Hospital of Nanchang University, Nanchang, Jiangxi, China; 2 196534 Department of Gastroenterology, Second Affiliated Hospital of Nanchang University, Nanchang, Jiangxi, China

**Keywords:** thrombin, thrombopoiesis, vasculopathies, pulmonary embolism

## Abstract

Dual inferior vena cava (DIVC) is a rare congenital anomaly complicating venous thromboembolism (VTE) management. This report describes a 59-year-old male with right lower extremity deep vein thrombosis and extensive pulmonary embolism. A DIVC was incidentally discovered during endovascular evaluation prompted by an atypical catheter trajectory, which precluded standard inferior vena cava filter placement. The patient was successfully managed with targeted catheter-directed thrombolysis for a significant embolic burden in the pulmonary artery. This case highlights the diagnostic and therapeutic challenges of DIVC, underscoring the need for a high index of suspicion for venous anomalies to guide individualized treatment in patients with extensive thromboembolic disease.

## Introduction

The inferior vena cava (IVC) is the primary conduit for venous return from the lower extremities and pelvis. Congenital IVC anomalies occur in 0.3 to 2% of the general population, with dual IVC being the most common. 1 Although often asymptomatic, these variants can be thrombotic, complicating interventions such as IVC filter (IVCF) placement. We present a case of pulmonary embolism (PE) in a patient with dual IVC, highlighting diagnostic and management challenges.

## Case Presentation

**Video 1** Inferior vena cava (IVC) angiography.


**Video 2** Pulmonary angiography.


**Video 3** Catheter-directed thrombolysis.


**Fig. 1:**
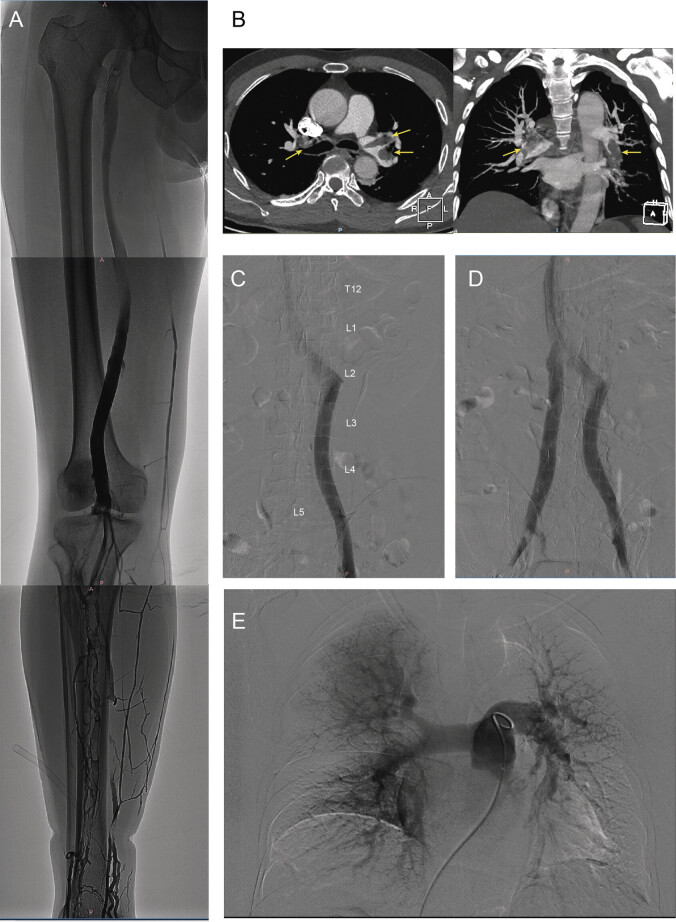
( **A** ) Antegrade venography of the right lower extremity deep venous system. ( **B** ) Computed tomography angiography (CTA) confirmed pulmonary embolism (PE) (the yellow arrow represents the thrombus). ( **C** ) Contrast-enhanced venography via left femoral catheterization revealed an anomalous left-sided inferior vena cava (IVC) positioned lateral to the vertebral column. ( **D** ) Bilateral iliac venography demonstrated a rare congenital anomaly: dual inferior vena cava (DIVC). ( **E** ) Pulmonary angiography was performed under digital subtraction angiography (DSA).

A 59-year-old male scheduled for right knee trauma surgery under general anesthesia was incidentally found to have hypoxemia (SpO _2_ 85% on room air) during preoperative evaluation. Notably absent were classic symptoms of PE such as chest pain or dyspnea. Physical examination revealed sinus bradycardia (42 bpm) and swelling of the right lower extremity. D-dimer was elevated (1,200 μg/L). Emergent point-of-care ultrasonography revealed deep vein thrombosis (DVT) involving the right gastrocnemius muscular vein and popliteal vein. Subsequent computed tomography angiography (CTA) of the pulmonary arteries confirmed multiple segmental emboli in both lungs ( Fig. 1B ).

The patient underwent combined deep venous and pulmonary angiography. Deep venography of the right lower extremity revealed DVT extending distal to the popliteal vein ( Fig. 1A ). During left femoral venous access establishment, anomalous vasculature was suspected due to atypical catheter trajectory ( Fig. 1C ). Bilateral iliac venography demonstrated a rare congenital anomaly: dual inferior vena cava (DIVC) with the left IVC originating from the left iliac vein (12 mm diameter) and the right IVC arising from the right iliac vein (13 mm diameter), both converging at the L1 vertebral level ( Fig. 1D ). Pulmonary angiography confirmed bilateral PE ( Fig. 1E ). The patient presented with peripheral deep venous thrombosis characterized by a low thrombus burden, and in the absence of clinical indications for prophylactic mechanical intervention, IVCF was not implanted. 2 3 However, given the identification of significant thromboembolic occlusion affecting the left lower lobar pulmonary artery, digital subtraction angiography (DSA)-guided endovascular catheter-directed thrombolysis was implemented as targeted interventional management to achieve localized fibrinolytic resolution of the critical embolic lesion ( see Videos 1 , 2 and 3 ).

## Discussion

DIVC arises from persistent embryonic supracardinal veins. 4 Due to venous stasis and associated endothelial dysfunction, DIVC predisposes individuals to DVT. 5 Underlying DIVC further exacerbates stasis and vascular wall injury, creating a prothrombotic environment. Studies indicate that approximately 5% of DVT cases are associated with venous malformations. 6 In this anomaly, thrombus from either iliac system may bypass the contralateral IVC, increasing PE risk even with unilateral DVT. The placement of an IVCF is a critical intervention for preventing PE, particularly in high-risk patients for whom anticoagulant therapy is contraindicated or has failed. 7 In this procedure, the anatomical characteristics of the IVC, especially its diameter, play a pivotal role in filter selection, placement safety, and long-term efficacy. An IVC diameter that is too small (e.g., <15 mm) may elevate the risk of filter penetration through the venous wall, particularly if the filter fails to fully deploy. 8 Traditional IVC filters are designed for single-lumen placement; dual IVC requires bilateral filters or advanced techniques, raising procedural risks. In this case, DIVC was detected through lower limb venography. CTPA alone may fail to identify DIVC. Therefore, abdominal/pelvic CT venography or magnetic resonance venography (MRV) should be considered in patients with idiopathic PE or when ultrasound findings are negative for DVT. 9

## Conclusion

DIVC, though rare, significantly impacts the management of thromboembolic disease. Critical care providers should maintain high suspicion for venous anomalies in bilateral DVTs or “high-volume” PEs. Individualized treatment strategy and comprehensive evaluation of the type of inferior vena cava malformation are necessary.

## References

[1] Spentzouris G Zandian A Cesmebasi A The clinical anatomy of the inferior vena cava: a review of common congenital anomalies and considerations for clinicians Clin Anat 2014 27 08 1234 1243 25042045 10.1002/ca.22445

[2] Kearon C Akl E A Ornelas J Antithrombotic therapy for VTE disease: CHEST guideline and expert panel report Chest 2016 149 02 315 352 26867832 10.1016/j.chest.2015.11.026

[3] Kitrou P M Katsanos K Papadimatos P Use of the Covera stent graft for the treatment of dysfunctional or thrombosed arteriovenous grafts: a retrospective analysis of 64 patients J Vasc Interv Radiol 2020 31 04 630 634 32127320 10.1016/j.jvir.2019.12.005

[4] Ang W C Doyle T Stringer M D Left-sided and duplicate inferior vena cava: a case series and review Clin Anat 2013 26 08 990 1001 22576868 10.1002/ca.22090

[5] Tritschler T Kraaijpoel N Le Gal G Wells P S Venous thromboembolism: advances in diagnosis and treatment JAMA 2018 320 15 1583 1594 30326130 10.1001/jama.2018.14346

[6] Li W R Feng H Jin L Chen X M Zhang Z W Duplication of the inferior vena cava: a case series J Int Med Res 2022 50 05):3000605221100771 10.1177/03000605221100771 PMC9134440 35607249

[7] Grewal S Lewandowski R J Ryu R KW Desai K R Inferior vena cava filter retrieval: patient selection, procedural planning, and postprocedural complications AJR Am J Roentgenol 2020 215 04 790 794 32755356 10.2214/AJR.19.22387

[8] Katyal A Javed M A Duplicate inferior vena cava filters: more is not always better Am J Emerg Med 2016 34 01 1150 1.15E6 10.1016/j.ajem.2015.04.083 25976267

[9] Milani C Constantinou M Berz D Butera J N Colvin G A Left sided inferior vena cava duplication and venous thromboembolism: case report and review of literature J Hematol Oncol 2008 1 01 24 19055711 10.1186/1756-8722-1-24 PMC2637295

